# Whole-Body Counter Evaluation of Internal Radioactive Cesium in Dogs and Cats Exposed to the Fukushima Nuclear Disaster

**DOI:** 10.1371/journal.pone.0169365

**Published:** 2017-01-18

**Authors:** Seiichi Wada, Nobuhiko Ito, Masamichi Watanabe, Takehiko Kakizaki, Masahiro Natsuhori, Jun Kawamata, Yoshio Urayama

**Affiliations:** 1 Laboratory of Veterinary Radiology and Radiation Biology, School of Veterinary Medicine, Kitasato University, Towada, Aomori, Japan; 2 Fukushima Headquarters for Animal Rescue, Fukushima, Fukushima, Japan; 3 Fukushima Veterinary Medical Association, Fukushima, Japan; Texas A&M University College Station, UNITED STATES

## Abstract

As a result of the 2011 nuclear incident that occurred at the Fukushima Nuclear Power Plant, a large number of abandoned dogs and cats were left within the disaster zone. A small number of these animals were rescued and cared for at shelters. Prior to the dispersal of these animals to their owners or fosterers, we evaluated the degree of internal radiocesium contamination using a specially designed whole-body counter. We conducted 863 non-invasive measurements of gamma rays due to internal radioactive cesium for 68 dogs and 120 cats at one shelter. After plotting graphs of ^137^Cs density we generated exponential functions of decay from seven dogs and six cats. From the regression formulae, we were able to determine the biological half-lives as 38.2 days for dogs and 30.8 days for cats. We found that in dogs there was a correlation between the biological half-life of radioactive cesium and age. Using our data, we estimated whole-body densities for each cat and dog at the time when they were rescued. We found that there were deviations in the data distributions among the different species, likely due to the timing of rescue, or living habits prior to rescue. A significant correlation was found when extracted feline reproductive organs were analyzed; the coefficients for the estimation of whole-body densities were approximately 7-fold higher than those based on the extracted feline reproductive organs. This may be due to the fact that majority of the radioactive cesium accumulates within muscular tissue with less distribution in other organs. It is possible to plan the appropriate management period in an animal shelter based on the use of the biological half-life of radioactive cesium calculated in this study. We believe that the correlations we uncovered in this work would be of great use for the management of companion animals in the event of a future nuclear accident.

## Introduction

On 11 March 2011, a major earthquake devastated the Tohoku region of Honshu Island of Japan, ultimately causing a nuclear meltdown in several nuclear reactors at the Fukushima Daiichi Nuclear Power Plant (FNPP). The large amounts of radioactive material that were released caused radioactive contamination throughout the Fukushima prefecture and surrounding areas. On 12 March 2011, evacuation orders were issued within a 20-km radius of FNPP. By 22 April 2011, the 20-km area was designated as a restricted zone, and the movement of animals and goods out of the area was highly restricted.

During this restriction, a ban was imposed on transportation of all livestock animals inside the restricted zone, while all companion animals (dogs and cats) were allowed out of the restricted zone after body surface radioactive contamination checking, as mandated by the Fukushima government.

There have been many analyses of human exposure to the release of radioactive materials into the environment from nuclear tests and atomic accidents. The primary focus of studies into animal exposure has been the transfer coefficients of radioactive materials to meats and milk [1, 2]. No data about domestically raised animals have been published. Prior to the FNPP accident, the 1986 Chernobyl nuclear accident exerted global influence on the handling of radioactive materials. Despite a large number of domestic meat animals being transported out of the restricted zone, all home breeding dogs around nuclear power plant were shot dead with no data on their internal contamination [3]. With regard to other wild animals, there is a large amount of published data on reindeer and roe deer from regions of Ukraine [4], on lynxes from northern Europe [5–7], and on wild boar from Germany [8]. Thus far, there are no reports pertaining to biological half-lives in animals, other than those that refer to the degrees of contamination or biodistribution.

In general, a multitude of different radioactive materials are released into the environment directly after a nuclear accident. Over time, changes occur within key nuclear species that can have an effect on human exposure. After several years, the nuclear species with the strongest effect are radioactive strontium and cesium. Because the amount of radioactive strontium emitted during the FNPP disaster was comparatively low, radioactive cesium was regarded as the greatest threat to human exposure in Japan [3, 9]. Consequently, radioactive cesium was selected to be the focus of this study. Additionally, the quality of life of domestic animals is taken into serious consideration in modern developed countries, and this is the second reason we focused our work on the biological half-life of radioactive cesium. Currently there are no data on cesium half-life in companion animals, yet this would form an important part in establishing the optimal management period for radioactively contaminated dogs and cats. Indeed, the provision of clean food and water for dogs and cats is a major issue for animal shelters. The longer that an animal stays in the shelter, the greater the probability that the shelter will be unable to provide for them adequately, due to increased numbers.

Although the Fukushima Headquarters for Animal Rescue initially established animal shelters at two locations, one shelter in Iino-machi, Fukushima, had closed by January 2013. The second shelter in Miharu-machi, in the Tamura district of Fukushima, was closed in December 2015 after operating for 4 years and 2 months. The majority of the animals being cared for at the Miharu shelter were dogs and cats that were rescued within the 20 km restricted area. These animals were treated before they were eventually transferred to owners or people who were willing to adopt them. Prior to the use of our whole-body radiation counter, to prevent exposure to the families who adopted the animals, we measured the radioactive dosage on each animal body surface using a survey meter and lengthened the raising period in the shelter for the attenuation of the internal radioactivity. Because the accurate biological half-life of radioactive cesium in cats and dogs is not known, we used parameters for humans. Although the biological half-life of radioactive cesium obtained from rodent and human data is applicable to other types of animals, there are presumably differences in metabolic rates between species. Additionally, given that many factors affect metabolic rates, it is critical to understand both the representative values and the margins of fluctuation.

To this aim, we installed a whole-body gamma counter at the Miharu shelter to monitor the amount of internal residual radioactivity in dogs and cats. Using this counter, measurements were collected from April 2013, for a period of 2.5 years. The effective half life in dogs and cats was measured in a prospective study and the relationship between the biological half-life and other factors was analyzed in a descriptive study. Data obtained non-invasively from dogs and cats that were exposed to a nuclear accident could be highly valuable. Indeed, our results should be useful for directing rescue activities of animal victims in the event of future nuclear accidents.

## Materials and Methods

### Animals and ethics

The Ethics Committee of Fukushima Headquarters for Animal Rescue at the Ministry of the Environment and Fukushima Veterinary Medical Association created the ethical standard manual for animal welfare (approved by an extraordinary meeting of Relief of Animals in Emergencies in May, 2011), studies were conducted in accordance with this manual. We obtained data for this research study at the Miharu shelter, which is a facility established for the humane rescue of dogs and cats affected by the earthquake that caused the Fukushima disaster. After the disaster, 463 dogs and 545 cats were collected from the restricted zone and taken to Miharu shelter, where veterinary care was carried out. The shelter also took responsibility for the acclimation of two to three new generations of animals born in the restricted zone since the disaster. Routine reports were given to multiple representatives from animal welfare groups and veterinarians about the operation and management of the shelter, the treatment of animals, and the collection of data on health management and exposure. Any necessary improvements were made in response to feedback.

At the Miharu shelter, the animals were housed in clean cages, at a controlled ambient temperature, and given appropriate food and water. To maintain health, animals underwent veterinary health check-ups (including fur checks), bathing, regular exercising, and were given toys to play with. During internal exposure measurements, the animals were housed in plastic cages suitable for their size, and then the measurements were performed non-invasively using a whole-body counter. To prevent breeding and other problematic animal behaviors, a skilled veterinarian castrated or spayed each animal immediately after its rescue. Almost all intact male and female cats underwent ovariohysterectomies and orchiectomies. The extracted reproductive organs were then measured for radioactivity to estimate the amounts of internal exposure.

### Whole-body counter

**Fig 1:**
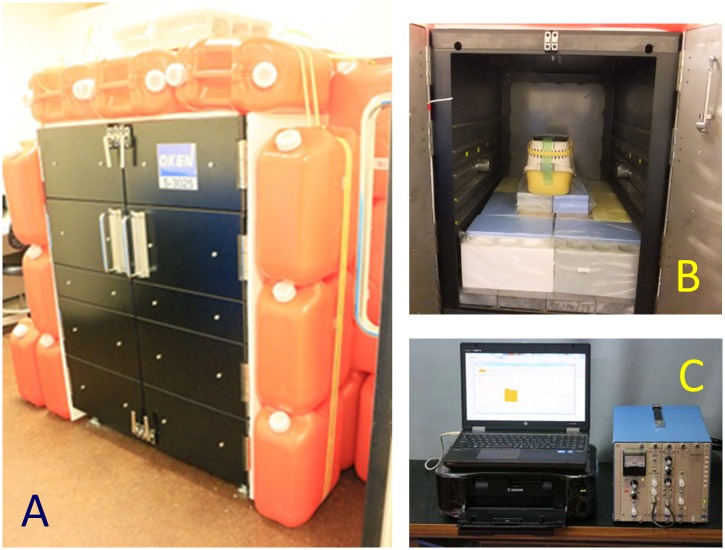
A: Front view of the detection tank of the whole-body counter. The entire surface, including the door, is covered with lead plates. The four vertical outer faces (front, back, left, right) were shielded with water. B: Inside the detection tank. The animal cage can be seen on top of polystyrene blocks. The NaI radiation detectors lie on both sides. C: High-voltage power supply, linear amplifier, multi-channel pulse height analyzer and radioactivity analyzer.

A whole-body counter was used to evaluate the internal exposure of rescued and acclimated animals. A total of 863 gamma ray measurements were performed non-invasively on 68 dogs and 120 cats at the Miharu shelter, to quantify the animals’ internal radioactive cesium. Measurements were conducted with the animal remaining inside their cage. The external dimensions of the steel detection tank were 880 mm (W), 1143 mm (D), 1225 mm (H) and 5 mm thickness. At the midpoint of the height of the 5-mm-thick steel detection tank, two 3 inch × 3 inch NaI (Tl) scintillation probes (Ohyo Koken Kogyo Co. Ltd., Tokyo, Japan) were installed. 15-mm-thick lead plates shielded all outer faces of the tank. To reduce background counts, the four vertical outer faces (front, back, left and right) were reinforced with a plastic container filled with 18 L of deionized water. They were set side by side and then fixed with cable ties to secure them in position (Fig 1).

The measuring instruments system consisted of a Nuclear Instrument Modules (NIM) standardized summation inverter, a linear amp, an Analog to Digital Converter (ADC) module, a high voltage power supply, a BIN power supply and a notebook PC with built-in analytical software. Ohyo Koken Kogyo Co. Ltd., Tokyo, Japan, manufactured the whole-body counter instrument. The instrument calibration was performed using a volume source with a ^137^Cs standard point source, and water contaminated from the nuclear accident as a reference volume source. The peak photoelectric absorption of ^137^Cs was selected at 661.66 keV energy and 85.1% branching rate of gamma rays, and calculations were performed. It is possible for the measured radioactivity to be affected by the influence of gamma ray emissions from ^134^Cs (796 keV), resulting in slight overestimations of radioactivity. The degree of the overestimations was confirmed to be less than 5% (at the time of instrument installation) by comparison to measurements of the calibration source by a germanium semiconductor HPGe detector (40% relatively efficient coaxial type; Canberra, Meriden, USA).

The animals were measured in a polypropylene cage that fit each animal’s individual physique and restricted their movements during measurements. Animals did not exhibit any violent or large movements during the measurements, meaning anesthetics or tranquilizers were unneeded. Prior to data collection, it was observed that the animals tended to sleep when the cage was darkened, so the decision was made to collect measurements with the lights switched off. In all instances, the measurements were performed in less than 1 h.

### Measurement of radioactivity of feline reproductive organs

The radioactivity of the surgically extracted reproductive organs was calculated via gamma ray spectrometry using a germanium semiconductor HPGe detector (GC3518; Canberra, Meriden, USA). In accordance with the sample radioactivity, the measurement times were between 3,600 and 200,000 s. The gamma ray energy efficiency was determined from measurements of standard point sources: ^133^Ba, ^137^Cs, ^60^Co, and ^22^Na. To derive the calculation efficiencies of the volumes of the measurement samples, solutions of potassium chloride containing known quantities of potassium were decanted into polyethylene vials (20 ml, 50 ml, and 100 ml) and multiple measurements were conducted on the volume sources. We obtained a calibration curve by evaluation of the photoelectric absorption peak of ^40^K for each of the 10 types of volume of potassium solution, and the measurement samples were corrected for volume efficiency. Corrections of decaying efficiencies were made on deviations from the ^40^K volume source. Numerical calculations were performed using the Simpson’s rule [10].

The samples were pretreated uniformly using a homogenizer and sealed in polyethylene vials (20 ml, 50 ml or 100 ml, according to their size); measurements were then performed. The limits of detection of radioactivity were determined from background counts.

### Statistical methods and analysis of biological decay of radiocesium

The measurements from the whole-body counter were recorded every few days until the internal radioactivity of each animal fell below the limits of detection. The limit of detection was 10–20 Bq/kg in a 1 hour measurement. The values from the first day of continuous measurements were normalized to be 1, and correlations between radioactivity and time were fitted as exponential functions of the logarithmic base. Regression equations and coefficients of determination were obtained using the least-squares method. For the correlation coefficients, the square roots of the determination coefficients were used, and for tests of no correlation, the Kolmogorov-Smirnov-test and the t-test were performed. The significant difference between ^137^Cs concentration from the whole-body of dogs and cats, and the genital organs of cats was tested using the Mann–Whitney U test, and for testing if there was no correlation, the Kolmogorov-Sminov test and the t-test were performed. Graphs were created using Microsoft Excel 2013 and statistical analyses were performed using R for Windows v.3.2.3. The effective half-life (Teff) was calculated from the regression equations of exponential decay from each animal, and the biological half-life (Tb) was obtained from the physical half-life of radioactive ^137^Cs (30.08 years) (Tp) and the effective half-life. Effective half-live was calculated by using the equation below.

$$\frac{1}{\text{Teff}}=\frac{1}{\text{Tp}}+\frac{1}{\text{Tb}}$$

## Results and Discussion

The Fukushima Headquarters for Animal Rescue have a policy to deliver disaster affected pet animals (rescued in the restricted zone or entrusted from the owner) to either their original owners or fosterers. To this end, a whole-body radiation counter was introduced, as it was essential to confirm the degree of internal exposure at the time when the animals were due to leave the animal rescue center. The use of whole-body counters for the evaluation of human internal exposure had already been widely used for this particular nuclear accident [9]; whereas the use of such instruments for animals had only been reported for evaluating radioactive cesium contamination in living beef cattle [11]. Although there have been examples of whole-body counters for dogs in studies on the effects of plutonium on living bodies [12] and in nuclear medicine [13], the Goiânia accident case was the only report we could find on the evaluation of internal contamination in living dogs [14]. We believe that this study is the first report on measurements of the elimination rates for radioactive cesium in pet dogs and cats using whole-body counters designed for animals.

**Fig 2:**
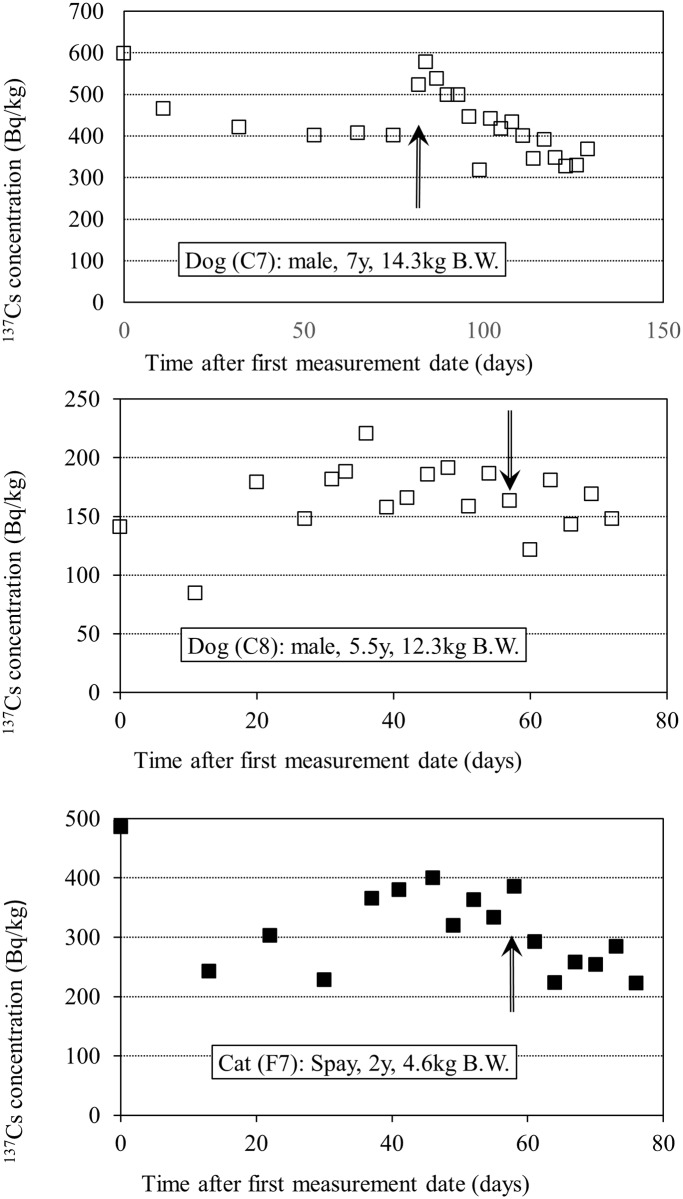
Observation of radiocesium decay after intervention. In these cases it was not observed radiocesium decay, so the animals’ bodies were washed again. The arrows indicate the point at which the animals were shampooed. C7 moved around in the cage a lot during the measurement, so its movement was limited by using a smaller cage and pillows.

Because clean food and water were given to the dogs and cats in the shelter, measurements were also taken for animals that had higher ^137^Cs levels than the limit of detection of the whole-body counter. We obtained high radiation counts for two dogs and one cat. However, the values did not change with time, so we resumed the measurements after washing the animals with shampoo and warm water. One of these dogs (C7) initially produced a high radiation measurement; C7 was restless inside the cage during these measurements, so we restricted the animal’s movement using a smaller cage and pillow, and observed a significant decline in measurements for C7 after this intervention. This was not seen in two further animals (C8 and F7) (Fig 2). Although we cannot deny the possibility that radioactive cesium was trapped within metabolically slow organs (such as the bones) of these animals, we surmised that because the phenomenon was only observed in those few animals, there was a miniscule amount of radioactivity fixed on their skin and fur. Using the animal data gathered from the decreasing trends over five repetitions, including the first measurements post-washing, we were able to obtain a significant exponential decay curve from the plotted densities of ^137^Cs for the 7 dogs and 6 cats.

**Fig 3:**
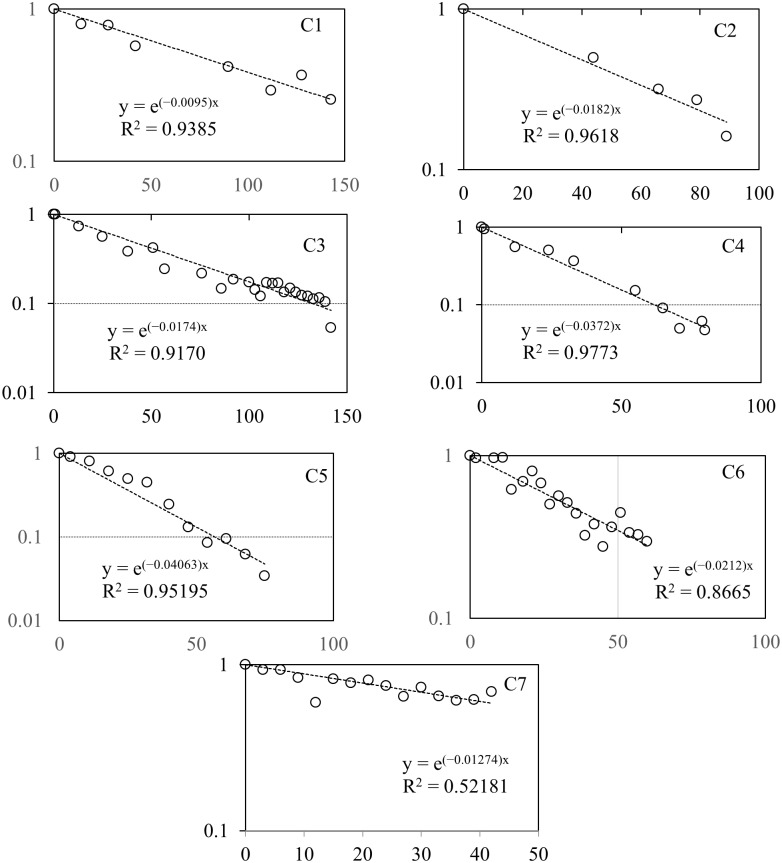
The decay curves of radiocesium (^137^Cs) in canine bodies. The graph shows the relative concentration in the whole body versus the number of days elapsed since the first measurement date. Each regression equation and the coefficient of determination are shown.

**Fig 4:**
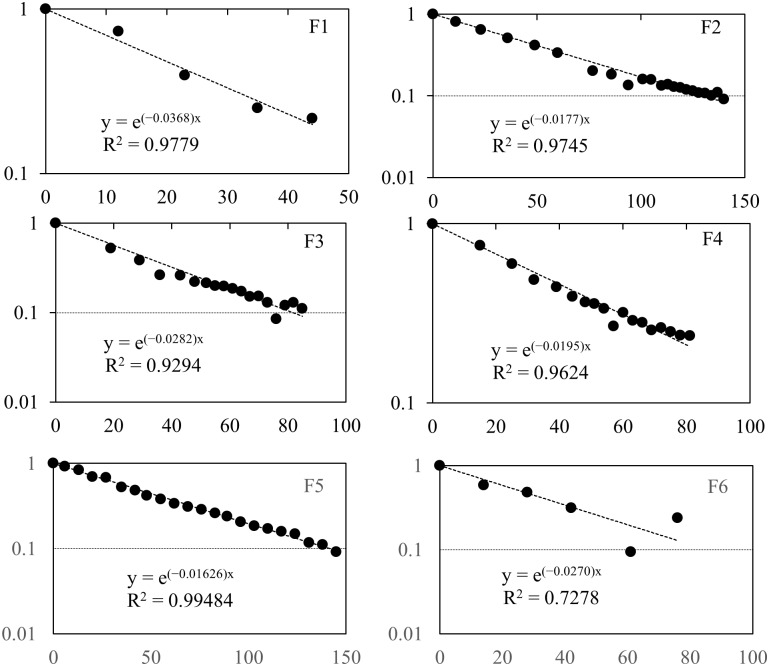
The decay curves of radiocesium (^137^Cs) in feline bodies. The graph shows the relative concentration in the whole body versus the number of days elapsed since the first measurement date. Each regression equation and the coefficient of determination are shown.

The regression equations and decay curves of the dogs and cats internal ^137^Cs densities are shown in Figs 3 and 4, respectively. The age, body weight, number of measurements, coefficients of determination for the regression formulae, significance levels of correlation, the effective half-lives of ^137^Cs and their median values, and the biological half-lives and their median values for the 7 dogs in Table 1 and 6 cats in Table 2 are shown. The individual breeds could not be identified since there was an abundance of mixed breeds.

**Table 1:** ^137^Cs biological half-lives and parameters of the decay curves of radiocesium in canine bodies.

Dogs	Sex	Age (years)	Body weight (kg)	Sample size (N)	Coefficient of determination (R^2^)	Significant level of correlation	Effective half life (days)	Biological half life (days)
C1	Castrated male	7.5	13.3	8	0.9385	p < 0.01	72.7	73.1
C2	Intact male	4.5	17.4	5	0.9618	p < 0.01	38.1	38.2
C3	Intact female	5.5	10.2	25	0.9170	p < 0.01	39.8	40.0
C4	Intact female	7.5	6.0	10	0.9773	p < 0.01	18.6	18.7
C5	Intact male	2	9.1	12	0.9520	p < 0.01	17.1	17.1
C6	Intact female	0.67	8.5	20	0.8665	p < 0.01	32.7	32.8
C7	Intact male	7	14.3	15	0.5218	p < 0.01	54.4	54.7
Median							38.1	38.2
Quartile 25%							25.7	25.7
Quartile 75%							47.1	47.3

**Table 2:** ^137^Cs biological half-lives and parameters of the decay curves of radiocesium in feline bodies.

Cats	Sex	Age (years)	Body weight (kg)	Sample size (N)	Coefficient of determination (R^2^)	Significant level of correlation	Effective half life (days)	Biological half life (days)
F1	Castrated male	3	4.76	5	0.9779	p < 0.01	18.8	18.9
F2	Spayed female	3	3.94	22	0.9745	p < 0.01	39.2	39.3
F3	Castrated male	7.5	4.28	24	0.9294	p < 0.01	24.6	24.6
F4	Spayed female	5	4.18	18	0.9624	p < 0.01	35.6	35.8
F5	Castrated male	5	4.68	24	0.9957	p < 0.01	42.8	43.0
F6	Spayed female	2	4.14	6	0.7278	p< 0.05	25.7	25.8
Median							30.7	30.8
Quartile 25%							24.6	24.6
Quartile 75%							38.3	38.4

The effective half-lives shown in Tables 1 and 2 were derived from the regression formulae of exponential decay shown in Figs 3 and 4. The biological half-lives were calculated from these effective half-lives. Given that the physical half-life of the target nuclear species ^137^Cs is 30.08 years, there were no major differences between the biological half-lives and the effective half-lives. In this study, the calculated biological half-lives of dogs showed a lot of variation, from 17.1 to 73.1 days (Table 1). In comparison, the upper and lower limits for the biological half-lives in cats were narrower, at 18.9 and 43.0 days, respectively (Table 2). However, large individual differences were still present. There were no significant differences in the median biological half-life values of dogs (38.2 days) and cats (30.8 days) (p = 0.39). In humans, the biological half-life of radioactive cesium is age-dependent and is longer for adults than for juveniles [15–17]. In one study, the average half-life of ^137^Cs in 23 Japanese adults was measured to be 86 days. In humans, the short half-life ^132^Cs demonstrates biphasic biological half-lives of 1–2 days for the first phase and 50–80 days for the second phase [18]. The existence of a third phase has also been suggested, based on analyses of individuals exposed during the Goiânia accident in Brazil [14]. In our study, however, we were unable to accurately demonstrate the existence of a second phase in dogs and cats. We believe this may be due to the dogs and cats at the Miharu shelter were not exposed to high levels of radioactive cesium.

There have been many studies on internal radioactive cesium contamination in wild animals [4–8] and on the transfer coefficients of the meat and milk from animal feed [1, 2]. Despite this, there have been very few studies that have focused on the biological half-lives of radioactive cesium scattered into the environment. Although there are detailed analytical studies on the internal radioactive distribution in cattle during the Fukushima nuclear accident [19, 20], there are few reports pertaining to the detailed metabolism of radioactive cesium [21]. A study was conducted where radioactive cesium-contaminated feed was given to cattle over a 4-month period, after which the contaminated feed was substituted with clean feed [21]. By evaluating the time-dependent biological half-lives in cattle urine, it was reported that the average biological half-life during a 0–9 day period was 7.1 days, while during a 44–128 day period an average of 37.4 days was obtained [21].

During our study, there was no observation of a biphasic exponential decay. We speculated that the majority of radioactive cesium in dogs and cats resides within the musculature, similarly to cattle [19, 20]. Based on the report of cattle exposure to high levels of cesium [21], it was thought that a bi- or tri-phasic decay pattern would be also observed in dogs and cats during a prolonged period of high level exposure. However, the non-contaminated food given to dogs and cats was provided by volunteer activists. Since this, the pollution level in dogs and cats in the shelter was not as high as that in the cattle.

**Fig 5:**
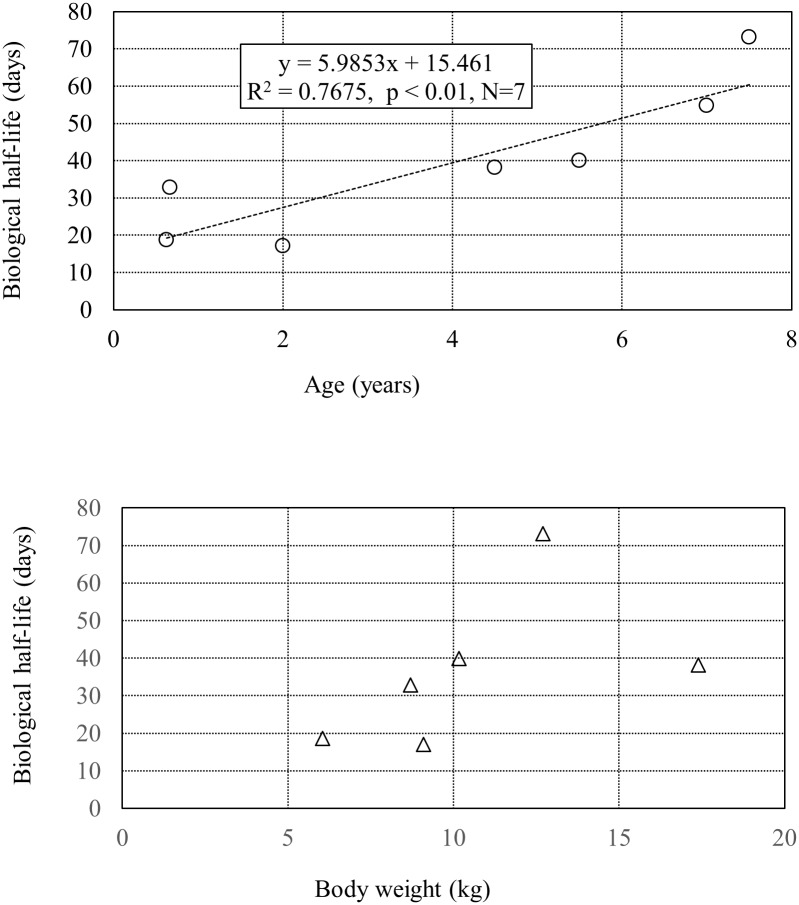
Correlation between canine biological half-life with age (top) and body weight (bottom). Regression equation, the coefficient of determination, and significant level of correlation are shown.

Because our study obtained the multiple decay curves of radioactive cesium in dogs and cats, we tested for correlations of the biological half-life of radioactive cesium with body weight or age. The results of the dogs are presented in Fig 5. The biological half-life demonstrates a significant correlation with age, showing that an age dependence is present in dogs. Although we also observe a tendency for the biological half-life to increase with body weight, no significant correlation was observed. Though not depicted in the figure, no relationship was observed between age, body weight, and biological half-life in cats. Because many of the cats were mostly similar in term of population characteristics (i.e., age and body weight) in this study, increasing the sample size may be necessary to probe the relationship between age, body weight and biological half-life. During the Goiânia accident in Brazil, Prussian blue (an ion-exchange drug) was administered to exposed individuals to encourage the excretion of radioactive cesium from the body. Reduction of the biological half-life has been reported in beagles (as an effect of the Prussian blue), and a correlation between body weight and biological half-life was also demonstrated [15]. Consequently, although it was anticipated that a positive correlation of age and body weight with biological half-life could be demonstrated for dogs—if detailed experiments were conducted on an increased number of individuals—from our data, we can only say that there is a correlation with age.

**Fig 6:**
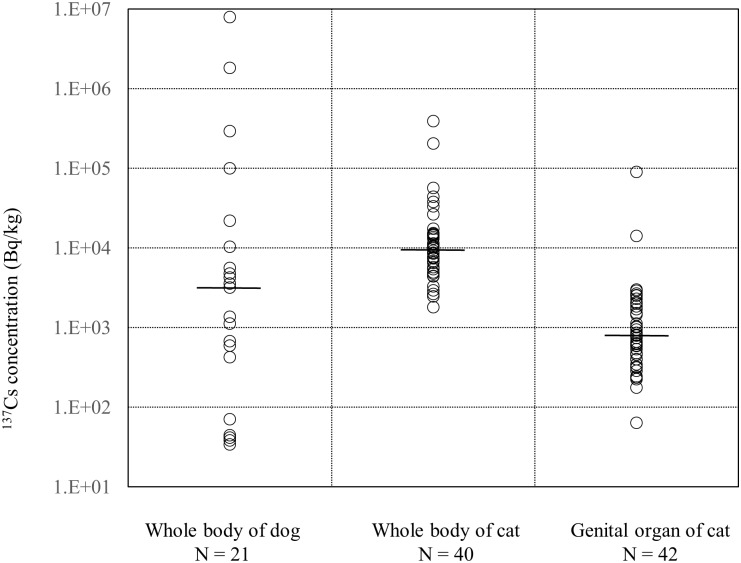
^137^Cs concentrations of canine whole-bodies, feline whole-bodies, and feline genital organs. The whole-body concentrations and genital concentrations were corrected to the time caught, using the effective half-life values. Horizontal short bars show the median.

Using the regression equation, which shows the correlations of biological half-life with age, each animal’s full body densities of radioactive cesium internal contamination at their time of capture were estimated for dogs and shown in Fig 6. These data are from 21 dogs and 40 cats, for which radioactive cesium was detected by the whole-body counter. Additionally, using the median biological half-life values, the individual whole-body densities estimated for cats, at their time of capture, are shown in Fig 6. The biological half-life values (C1–C7, F1–F6) used for the calculation in Fig 6 were the data shown in Tables 1 and 2. Furthermore, to curb the problem behaviors accompanied with breeding behavior, both castration and spaying were carried out on the cats. The corrected radioactive cesium densities of the extracted reproductive organs at the time of capture, similarly to the whole-body measurements, are shown in Fig 6.

From Fig 6 the differences in the data distribution of whole-body concentration between the dogs and cats can be clearly observed. The median whole-body value for the cats was higher, at 9,520 Bq/kg, than for the dogs, at 3,160 Bq/kg, and this was a significant difference (p<0.01). In addition to this, from the lowest and highest individual densities in dogs, it can be seen that the distribution range was wide, with a 100,000-fold difference. For cats, this distribution range was narrower, with the highest value only 100 times larger than the lowest. The order of the median values for both dogs and cats was on the same power of 10, and while we thought no significant difference in the median values, we believed that there are reasons for the differences in the whole-body data distributions. The first reason is the capture timing and duration of the holding period for the animals’ rescue. Among the 40 cats in which radioactive cesium was detected via whole-body counter, the capture timing for 37 cats was concentrated within a 100-day period from 13 September 2012 to 21 December 2012. For the 21 dogs, there was a 3.5-year capturing period, from 5 June 2011 to 12 December 2014. The radiocesium pollution on the soil surface of the restricted zone is expected to decrease with time, in particular the radiocesium pollution of the city environment is expected to decreased faster than that of the forest environment; therefore, the animals that live there are considered to have less internal exposure as time passes. The second reason is due to the presumed differences in behavior between dogs and cats. At the time when the dogs were rescued, all of them roamed wild outdoors. We assumed that since their range of activities was wider and the consumption of water was larger, the internal radioactive concentrations for dogs were a reflection of the contamination in their individually marked territories. Their food was provided by volunteer activists early on in the rescue process, but their drinking water was primarily rain and standing water. In contrast, the cats had lived indoors, only occasionally venturing outside, and so their rescue activities were conducted mainly within human habitations. We surmised that due to this, their habitats might be collectively more similar to human habitats, and thus have a comparatively narrow concentration range. In reality, compared with the dogs, the capture of the cats was more difficult, and because a focused capture of the cats was conducted, it was considered that the distribution of pollution of in cats was not definitively observed.

**Fig 7:**
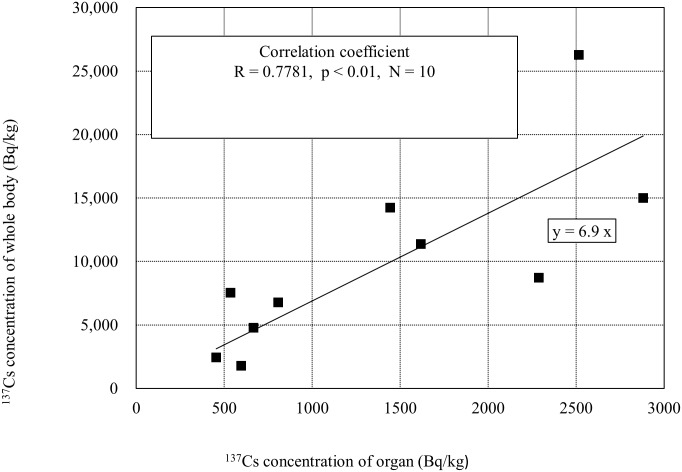
Correlation between whole-body radioactivity and genital organ radioactivity in cats.

The distributions of radioactive cesium densities for the feline reproductive organs are shown in Fig 6. The median value is 790 Bq/kg, which shows a significant difference from the cats’ whole-body densities (p<0.01). We were able to obtain corresponding data on the whole-body densities and reproductive organ densities for 10 of the cats, and this relationship is plotted in Fig 7. From this, we see that the whole-body densities are higher than the reproductive organ densities, with a significant correlation. Owing to the analysis of internal organs and muscle tissue of cattle contaminated during the FNPP accident, it is already known that the majority of radioactive cesium accumulates in the muscles [19, 20], and very little is distributed to other organs. We assumed that even for cats, the whole-body densities would be high. The whole-body densities of the 10 cats in Fig 7 were roughly 7 times the reproductive organ densities.

Our whole-body counter, designed specifically for the dogs and cats in this study, was installed at the Miharu shelter 2 years after the FNPP accident; if it had been installed at an earlier stage more precise high quality information could have been acquired. Radioactive cesium taken into the animal body is discharged to the outside of the body by giving them clean food and water. By means of the biological half-life of radioactive cesium in this study, it is possible to plan the appropriate management period in an animal shelter. Optimizations of the animal management period contribute to animal welfare as well as reduction in administration expenses. We were able to survey the biological half-life of radioactive cesium in dogs and cats contaminated by an actual nuclear accident. We believe that our findings on biological half-life in dogs and cats are valuable. We are confident that this information is useful for the management of domestic animals in the event of a future nuclear accident.
